# Granzyme A Produced by γ_9_δ_2_ T Cells Activates ER Stress Responses and ATP Production, and Protects Against Intracellular Mycobacterial Replication Independent of Enzymatic Activity

**DOI:** 10.3389/fimmu.2021.712678

**Published:** 2021-08-03

**Authors:** Valerio Rasi, David C. Wood, Christopher S. Eickhoff, Mei Xia, Nicola Pozzi, Rachel L. Edwards, Michael Walch, Niels Bovenschen, Daniel F. Hoft

**Affiliations:** ^1^Department of Molecular Microbiology and Immunology, Saint Louis University School of Medicine, Saint Louis, MO, United States; ^2^Department of Internal Medicine, Saint Louis University School of Medicine, Saint Louis, MO, United States; ^3^Department of Biochemistry and Molecular Biology, Saint Louis University School of Medicine, Saint Louis, MO, United States; ^4^Anatomy Unit, Department of Oncology, Microbiology and Immunology, Faculty of Science and Medicine, University of Fribourg, Fribourg, Switzerland; ^5^Department of Pathology, University Medical Center Utrecht, Utrecht, Netherlands; ^6^Center for Translational Immunology, University Medical Center Utrecht, Utrecht, Netherlands

**Keywords:** Granzyme A, *Mycobacterium tuberculosis*, BCG, ER stress response, ATP production, 2D-DIGE, human monocyte, GZMA

## Abstract

*Mycobacterium tuberculosis* (Mtb), the pathological agent that causes tuberculosis (TB) is the number one infectious killer worldwide with one fourth of the world’s population currently infected. Data indicate that γ_9_δ_2_ T cells secrete Granzyme A (GzmA) in the extracellular space triggering the infected monocyte to inhibit growth of intracellular mycobacteria. Accordingly, deletion of *GZMA* from γ_9_δ_2_ T cells reverses their inhibitory capacity. Through mechanistic studies, GzmA’s action was investigated in monocytes from human PBMCs. The use of recombinant human GzmA expressed in a mammalian system induced inhibition of intracellular mycobacteria to the same degree as previous human native protein findings. Our data indicate that: 1) GzmA is internalized within mycobacteria-infected cells, suggesting that GzmA uptake could prevent infection and 2) that the active site is not required to inhibit intracellular replication. Global proteomic analysis demonstrated that the ER stress response and ATP producing proteins were upregulated after GzmA treatment, and these proteins abundancies were confirmed by examining their expression in an independent set of patient samples. Our data suggest that immunotherapeutic host interventions of these pathways may contribute to better control of the current TB epidemic.

## Introduction

*Mycobacterium tuberculosis* (Mtb), the etiological agent that causes tuberculosis (TB), is the number one infectious killer worldwide causing 1.4 million deaths in 2019 alone (1). In recent years, there has been an increase in the number of multidrug resistant (MDR), as well as extensively drug resistant (XDR) TB cases, highlighting the urgent need to develop new therapeutics to eradicate this disease. While several clinical trials have evaluated host-directed therapies for TB (2), current treatment regimens only target antimicrobial pathways, which engenders antimicrobial resistance to emerge.

Despite WHO eradication efforts, the only licensed TB vaccine is the Bacillus Calmette–Guerin (BCG), which is an attenuated strain of *Mycobacterium bovis* that causes disease in cattle. While the BCG vaccine protects children from acute meningitis and miliary TB, it is less protective against adult pulmonary disease and aerosol transmission. Therefore, new strategies must be employed, including novel immunotherapies that trigger host responses capable of inhibiting mycobacterial growth *in vivo*.

Our lab has previously shown that γ_9_δ_2_ T cells develop a memory response after BCG vaccination (3), potently inhibit the intracellular replication of mycobacteria (4), and produce Granzyme A (GzmA), a key mediator for this inhibition (5). Since mice lack γ_9_δ_2_ T cells specifically, studies are underway in non-human primates (NHP) to evaluate the protective role of γ_9_δ_2_ T cells *in vivo*. Further, data indicate that Mtb-derived 6-O-methylglucose–containing lipopolysaccharides (mGLP) induces potent γ_9_δ_2_ T cell-expansion, higher frequencies of γ_9_δ_2_ T cells producing GzmA, and mycobacterial inhibition (6). Preliminary data suggest that rhesus macaques vaccinated with mGLP have lower bacterial load at the site of inoculation, no dissemination to other lung lobes, and lower pathological disease burden than controls (manuscript in preparation). Thus, γ_9_δ_2_ T cells are an attractive target for novel TB vaccine design and GzmA-mediated mechanism of inhibition requires more investigation.

GzmA is a serine protease released from secretory granules by activated NK cells and T cells. Recently, GzmA has been shown to induce a pro-inflammatory profile in monocytes and macrophages (7–11). Our group has previously shown that γ_9_δ_2_ T cells secrete GzmA upon contact with mycobacteria-infected macrophages and importantly concentrations of GzmA correlate with mycobacterial inhibition. Accordingly, *GZMA* gene knockdown in γ_9_δ_2_ T cell clones reversed this inhibitory activity whereas native human GzmA added exogenously to infected macrophages leads to inhibition of intracellular mycobacterial growth (5). Transcriptional analyses have failed to uncover the pathways responsible for mycobacterial growth inhibition. However, due to the protease activity harbored by this family of enzymes, GzmA-mediated control of intracellular mycobacterial was postulated to occur at the protein level.

Global proteomics experiments were performed to elucidate how GzmA activates monocytes to kill intracellular mycobacteria. We report here that GzmA added to BCG-infected monocytes activates the ER stress response and ATP producing proteins and leads to intracellular inhibition of mycobacteria. Site-directed mutagenesis demonstrates that enzymatic activity is not necessary to mediate mycobacterial growth inhibition. Since GzmA is internalized in infected cells, we speculate that key features within the protein structure activate target cells to induce the ER stress response and ATP-producing proteins to control mycobacteria infection.

## Materials and Methods

### Human Samples

Healthy adult volunteers were recruited according to protocols approved by the Saint Louis University Institutional Review Board #26646 and #26645. Written consent from the volunteers was obtained according to the principles expressed in the Declaration of Helsinki. Ficoll-Paque (GE Healthcare, Piscataway, NJ) was used to obtain PBMC from leukapheresed samples. Adherent monocytes were isolated by plastic adherence as previously described (12).

### Preparation of Mycobacteria Infection

Connaught strain BCG was grown to mid-logarithmic phase in Middlebrook 7H9 media supplemented with 10% albumin, dextrose, catalase (ADC; Cat # 211887 BD Diagnostics, Franklin Lakes, NJ) + 0.05% Tween-80. Stocks were aliquoted in media without Tween -80 and frozen at -80°C. The concentration of the bacterial stock was determined after thawing by CFU plating performed in triplicate. Thawed aliquots were sonicated to generate single-cell suspensions before dilution and infection of monocytes.

### Mycobacterial Growth Inhibition Assay (MGIA)

The assay was performed as previously published with slight modifications (12). Briefly, thawed PBMC were plated on round-bottom 96-well plates in RPMI-1640 (Gibco Cat #11875, Thermofisher, Wantham, MA) supplemented with 10% Human AB serum (Sigma, St. Louis, MO) and 1% L-glutamine (Sigma, St. Louis, MO) (without antibiotics); complete media is termed R+2. After 2 h, cells were gently washed with warmed R+2 media at 37°C to remove non-adherent cells. The monocytes (mostly CD14^+^) attached to the plate were then infected with Connaught BCG (Multiplicity of Infection=3) and treated with 200 nM Granzyme A (GzmA). After 1 h, cells were gently washed with R+2 media three times to remove extracellular BCG and resuspended in R+2 media containing 200 nM GzmA. After 72 h co-culture, cells were lysed 0.2% saponin (Cat # S7900, Sigma, St. Louis, MO) solution in RPMI-1640, and the reaction quenched after 2 h with 100 µL 7H9+ADC containing 1 µCi 5,6-^3^H-uridine. After 72 h, plates were harvested onto glass fiber filter papers (filtermats). Filtermats received Illumina Gold F scintillation fluid (Cat # 6013321, PerkinElmer, Waltham, MA) and were imaged using a MicroBeta^2^ liquid scintillation counter (PerkinElmer, Waltham, MA) measuring Disintegration Per Minute (DPM). The % inhibition was calculated as: 100 – 100 x (DPM from wells treated with GzmA and infected with BCG/DPM from wells infected with BCG).

### GzmA Purification

Native GzmA was purchased from TheraTest (Lombard, IL), which utilized previously published techniques (13). Recombinant GzmA was purified after transient transfection of HEK293T cells (ATCC^®^ CRL­11268TM; ATCC, Manassas, VA) with *GZMA* encoded within the pHL-sec plasmid (14). Dr. Walch kindly provided the plasmids that were then amplified in endotoxin-free Giga-kits (Qiagen, Germany). GzmA-S195A was obtained by submitting GzmA-WT pHL-sec plasmid to Genewiz (South Plainfield, NJ), which performed site directed mutagenesis at position 212 (195 using tryptase numbering) and substituted Ala to Ser. Genewiz also verified successful mutagenesis and released report. For RhGzmA-WT and Rh-GzmA-S195A purification, HEK293T cells were incubated at 37°C for 7-11 h with plasmid in FBS-containing DMEM media using Lipofectamine 3000 [instead of Calcium Phosphate transfection as in (14)]) and following the recommended concentrations (Cat # L3000008, Thermofisher, Waltham, MA). Supernatant-containing GzmA was then harvested 72-96 h after transfection [improved method by not switching to serum-free media compared to (14)]. The purification protocol was modified to maintain buffers in either isotonic or hypertonic solution, as protein precipitation occurred in hypotonic buffers. Purification of supernatant was performed at 4°C first using Ni-IMAC column (Cat # 17531806, GE Healthcare, Chicago, IL), activation of GzmA after enterokinase (Cat # SRP3032, Sigma, St. Louis, MO) treatment at room temperature (RT), and final MonoS column (Cat # 17516801, GE Healthcare, Chicago, IL) purification at 4°C. All steps were completed with single-use plastic bottles and endotoxin-free reagents and the final protein was run over an Endotrap column (Lionex Gmbh, Germany) to remove any residual endotoxin contamination. Homodimerization was verified by non-reducing SDS gel electrophoresis, and protein was verified by western blot with a GzmA 1:500 antibody (R&D, Clone #356422) and further, by the lack of interaction with Granzyme K 1:100 antibody (Cat # SAB2103935, Sigma, MO). WT and GzmA-S195A were tested using MALDI-TOF, which confirmed protein purification as well as site-directed mutagenesis (data not shown). Protein was stored at -80°C in 10 µL aliquots and thawed and diluted the day of the experiment.

### BLT Esterase Assay

The substrate Z-L-Lys-SBzl hydrochloride (Sigma, St. Louis, MO) was added at different concentrations (19.5-2,500 µM to measure Vmax and Km, and 2,500 µM for GzmA-S195A experiments) and diluted in assay buffer (50 mM Tris, 154 mM NaCl, pH 7.5) in presence of 0.55 M (5,5-dithio-bis-(2-nitrobenzoic acid) (DTNB) (Sigma, St. Louis, MO) chromophore. 120 pM of protein was added per well and substrate hydrolysis was quantified by measuring the absorbance at 405 nm using an SLT Rainbow plate reader (Tecan, Männedorf, Switzerland). Esterolytic activity was reported as rate of hydrolysis using extinction coefficient of 13,100 M^-1^cm^-1^ for the 3-carboxy-4-nitrophenoxide ion. Specific activity measured as nM product/min/nM of enzyme present.

### DCI Experiments

150 µM of 3,4-dichloroisocoumarin (DCI) (Cat# D7910, Sigma, St. Louis, MO) was incubated with GzmA (7.5 µM) for 30 min at RT. DCI-treated GzmA was then dialyzed against 50 mM Hepes, 150 mM NaCl, pH 7.4, and its inactivation was confirmed by BLT assay.

### GzmA Internalization and Confocal Microscopy Experiments

To monitor GzmA internalization, monocytes were first blocked with True Stain Fc blocker (Cat. # 426101, Biolegend, San Diego, CA), and then incubated with anti-GzmA antibody conjugated with PE (Cat. #558904, BD Biosciences, San Diego, CA). Permeabilization solution was used to study GzmA internalization (Cat. #554715, BD Biosciences, San Diego, CA). For confocal microscopy experiments, cells were incubated on Lab-tek tissue culture-treated wells (Cat. #154941, Nunc, Denmark). At the time of fixation, cells were washed twice with 1x RT PBS, and fixed in 4% paraformaldehyde for 15 min at 37°C. Then slides were washed thrice for 5 min with RT 1x PBS, and then blocked with 5% donkey serum [52-000-121] with 0.25% Tween-20 (Cat. #P9416, Sigma, St. Louis, MO). Cells were then incubated overnight at 4°C with primary antibody anti-GzmA (Cat. # MAB29051, R&D, Minneapolis, MN) at 1:20 (12.5 µg/mL) in blocking buffer. To remove unbound antibody, cells were washed four times with 1x PBS at RT, and then incubated in the dark for 1 h with secondary antibody conjugated with Alexa Fluor 594 (Cat. # 715-545-150, Jackson Immunoresearch, West Grove, PA) at 1:100. Cells were then washed thrice with 1x PBS at RT and incubated with 2.86 µM DAPI (Cat. #5748, Tocris, Bristol, UK) for 5 min at RT. Slides were finally mounted using Prolong Diamond antifade mountant (Cat. #P36965, Life Technologies, OR, USA) overnight, and then stored at -20°C until visualized using instrument at Saint Louis University Research Microscopy Core.

### 2D DIGE Analysis and MALDI-TOF

Cells were washed twice with sterile 1x PBS, then lysed in buffer compatible for 2D separation (8 M urea, 2 M thiourea, 4% CHAPS, 50 mM Tris, pH 8.5) in presence of 1:100 HALT phosphatase and protease inhibitor (HALT phosphatase and protease inhibitor (Cat # 78442, Thermo-Fisher, Waltham, MA). Samples underwent ReadyPrep 2Dclean-up kit (BioRad, Hercules, CA). Protein concentrations were quantified using Pierce 660 nm protein assay (Cat # 23236, Thermo-Fisher, Waltham, MA). Prior to carrying out DIGE substoichiometric CyDye labeling, an equal protein quantity was taken from each lysate to create a pool for normalization of fluorescence intensity for all analytical 2D gels. This pool was labeled with Cy2 dye, and individual samples were labeled with either Cy3 or Cy5 (Lumiprobe, Hunt Valley, MD) using a dye ratio of 8 pmol per µg protein. Samples were incubated on ice in dark for 30 min. The reaction was quenched by adding 10 mM lysine and incubating on ice for 10 min. Samples were pooled (Cy2, Cy3, and Cy5) on a immobiline dry strip immobilized pH gradient (IPG) pH 3-11 Non-Linear (NL) (GE Healthcare, Chicago, IL), 7 cm, rehydrated overnight in rehydration buffer (30 mM Tris, pH 8.5, 2 M thiourea, 7 M urea, 2% dithiothreitol (DTT), 4% CHAPS, and 0.5% IPG buffer) at 30 V. Isoelectric Focusing (IEF) separation was performed according to manufacturer instructions (total 11 kW). IEF strip was reduced in 10 mg/mL DTT and then alkylated in 25 mg/mL iodoacetamide in reducing buffer (100 mM Tris, pH 8.8, 6 M urea, 30% glycerol, 2% SDS). Proteins were separated by SDS-PAGE using Criterion XT 4-12% SDS-PAGE gels (BioRad, Hercules, CA) and applying 100 V for 45 min. Gels were fixed in 30% methanol, 7% acetic acid, and then washed twice in distilled water. CyDyed spots were detected with a Typhoon 9410 scanner (GE Healthcare, Chicago, IL) with PMT voltage adjusted to a maximum intensity between 80,000-95,000 and with <15% variation between Cy2, Cy3, and Cy5. Melanie software V.9.1.1 (GE Healthcare, Chicago, IL) analysis was used to overlap gels from different subjects and conditions to find identical protein spots across gels, normalization of Cy3 and Cy5 to Cy2, and fold change of relative abundance after BCG infection and/or GzmA treatment. The average fold change of a spot in two subjects that was >1.3 in Group 4 relative to Group 3 and Group 1 was isolated for identification. Spots corresponding to differentially abundant proteins were identified using MALDI-TOF mass spectrometry. Following in-gel tryptic digestion (15), we used an Axima Resonance MALDI-TOF mass spectrometer (Shimadzu, Kyoto, Japan) to identify the differentially abundant protein spots. Spots that had a MALDI score of at least 60 were chosen indicating that there is less than 1 in 1e^6^ chances that this protein was discovered by a random coincidence.

### Protein-Protein Interaction Network

To construct protein-protein interactions for GmzA-associated proteins, the Search Tool for the Retrieval of Interacting Genes/Proteins (16) database version 10.5. was used (17).

### Statistical Analysis

For generation of graphs and statistical analysis we used GraphPad Prism version 9.0.0 for Windows, GraphPad Software, San Diego, California USA, www.graphpad.com.

## Results

### Recombinant Human GzmA Phenocopies Native Protein

**Figure 1:**
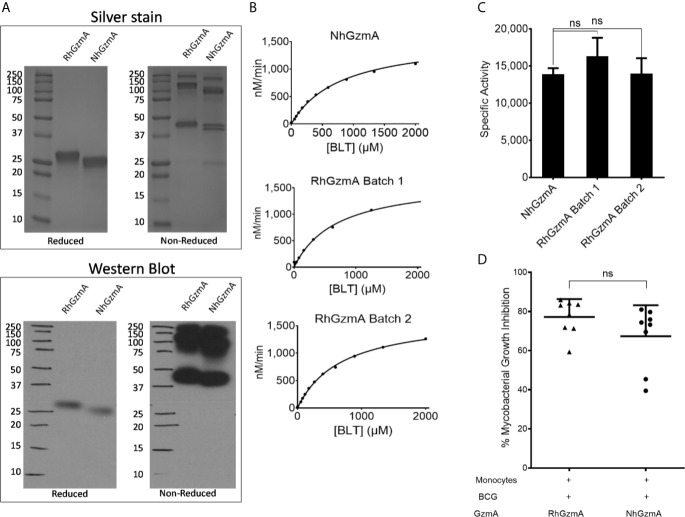
Recombinant human GzmA phenocopies native protein. **(A)** RhGzmA and NhGzmA homodimerize (~50 kDa, some monomer at ~25 kDa in NhGzmA) and are pure as shown by silver stain (top figure) and western blot (bottom figure). Under reducing conditions, GzmA assumes the monomer form, while under non-reducing conditions homodimer and polymers of GzmA are the predominant forms. RhGzmA has a His-tag at the C-terminus of the protein, which accounts for the slightly heavier MW. **(B, C)** RhGzmA and NhGzmA cleave BLT substrate at similar rates, and different lots of RhGzmA have similar enzymatic activity in the BLT esterase assay. Results are plotted using the Michaelis-Menten equation **(B)** and fitted to produce specific activity **(C)**. Commercially obtained native protein, and two different lots of purified recombinant protein are shown. Negative control results (BLT alone) were subtracted from results for each experiment (unpaired t-test, experiment repeated at least three times). **(D)** Recombinant human GzmA recapitulates mycobacterial growth inhibition as native protein measured in the Mycobacterial Growth Inhibition Assay (n=8; data representative of two independent experiments; means and SEM; Wilcoxon matched-pairs signed rank test) (*p < 0.05, **p < 0.01, ***p < 0.001; ns, not significant).

Our previous studies that confirmed the mycobacterial growth inhibition in infected macrophages were conducted using native human GzmA (NhGzmA). However, due to the low yield (50 μg from the purification of 1x10^9^ NK92MI cells-data not shown) that is obtained and the potential co-elution with other granzymes, we decided to compare commercially available NhGzmA to recombinant human (RhGzmA). RhGzmA was purified from transiently transfected HEK293T cells with an improved purification method that allowed us to produce 20 mg of highly purified GzmA protein, which is 10 fold higher than previously reported methods (14), and can be utilized for large global proteomic studies. RhGzmA was shown to form homodimers (~50 kDa) similar to NhGzmA by silver stain and western blot (**Figure 1A**) as well as polymers of the 50 kDa protein as previously reported (11). RhGzmA has a slightly higher molecular weight because of the presence of a His-tag at the C-terminus of the protein. To compare the specific enzymatic activities of recombinant and native GzmA, we analyzed their ability to cleave the chromogenic substrate, Z-L-Lys-SBzl hydrochloride (BLT). Data indicate that the recombinant protein is similar to the native protein in that both can efficiently cleave BLT (**Figure 1B**). It is important to note that substrate cleavage efficiencies were found to be similar between independently purified batches of recombinant GzmA, demonstrating the reproducibility of our purification method (**Figure 1C**). To confirm whether RhGzmA and NhGzmA induce similar protective effects against mycobacteria, monocytes were infected with BCG and treated with either recombinant or native human GzmA and the degree of mycobacterial growth inhibition was measured. As expected, RhGzmA inhibited almost 80% of the intracellular replication of mycobacteria, and no significant difference was detected between the recombinant protein and the native form (**Figure 1D**).

To verify that the inhibitory activity is independent of endotoxin, RhGzmA was heated at 95°C for 10 min to denature the recombinant protein while retaining the activity of a potential endotoxin contaminant as reviewed in (18). The ability of the heat-treated protein was then assayed for its ability to inhibit mycobacterial growth. As demonstrated in **Supplementary Figure 1A**, heat denaturation of RhGzmA abolished the intracellular growth inhibition suggesting that endotoxin contamination did not explain the protective effects of RhGzmA. To further rule out whether endotoxin contributes to the robust growth inhibition displayed by the recombinant protein, RhGzmA was passed through an Endotrap column to remove any residual endotoxin and then tested for its ability to inhibit growth. Data indicate that when purified RhGzmA is treated for endotoxin removal, the protein retains its capacity to restrict intracellular growth (**Supplementary Figure 1B**). Taken together, these data indicate that RhGzmA recapitulates the effects of native GzmA, and accordingly, can be used for large scale applications such as global proteomic studies to help identify molecular mechanisms of protection.

### Enzymatic Activity Is Dispensable to Mediate Mycobacterial Growth Inhibition

**Figure 2:**
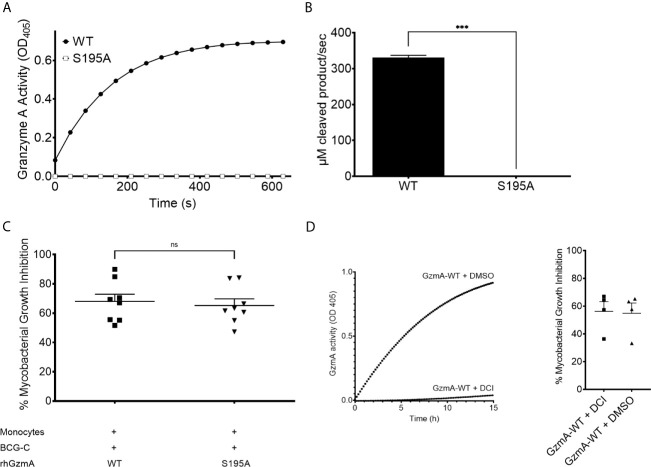
Enzymatic activity is dispensable to mediate mycobacterial growth inhibition. **(A)** GzmA of wild-type (WT) and S195A variant proteins’ activity over time measured by the BLT assay of three independent experiments performed as triplicates per condition. **(B)** For statistics, the rate of initial reaction was compared between WT and S195A variant (means and SD; paired t-test). **(C)** MGIA comparing WT and S195A enzymatically inactive variant, displayed as % of inhibition (n=8 in at least two independent experiments. Mean and SEM, Wilcoxon matched-pairs signed rank test). **(D)** GzmA treated with DCI inhibits intracellular replication as GzmA-DMSO in MGIA (left) and BLT assay (right) showing that inhibited GzmA retains inhibitory activity (n=4, Mean and SEM) (*p < 0.05, **p < 0.01, ***p < 0.001; ns, not significant).

GzmA is a trypsin-like serine protease, cleaving substrates after positively charged lysine and arginine residues (19). GzmA possesses the canonical serine protease catalytic triad formed by His57, Asp102, and Ser195. To determine whether its enzymatic activity is necessary to mediate mycobacterial growth inhibition, Ala was substituted for Ser195 and the protein variant was purified similar to the wild-type (WT) RhGzmA (**Supplementary Figure 2**) (10, 11, 20). The S195A mutant was then evaluated for its ability to cleave BLT. The single amino acid substitution abolished catalytic activity (**Figures 2A, B** **)**. The catalytically inactive S195A GzmA protein was then tested in the mycobacterial growth inhibition assay (**Figure 2C**), which revealed similar mycobacterial inhibitory activity comparing WT and S195A, implying that a catalytically active enzyme is not required to inhibit intracellular growth. To confirm that catalytic activity is unnecessary for mycobacterial growth inhibition, WT GzmA was incubated with the serine protease inhibitor 3,4 Dichloroisocoumarin (DCI), and then tested for its ability to restrict growth. While WT GzmA treated with DCI was unable to cleave the BLT substrate (**Figure 2D**) similar to the S195A mutant (**Figure 2A**), its ability to mediate mycobacterial growth inhibition was not affected indicating that enzymatic activity is dispensable (**Figure 2D**).

### GzmA Is Internalized in Infected Monocytes

**Figure 3:**
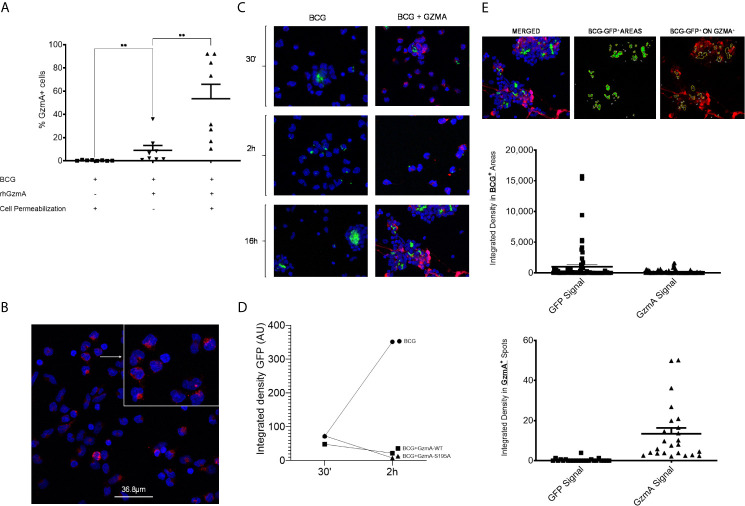
GzmA is internalized in infected monocytes. **(A)** In flow cytometry studies comparing surface *vs.* intracellular staining, the majority of GzmA is inside the monocytes after treatment (n=8 in at least two independent experiments; means and SEM; Wilcoxon matched-pairs signed rank test). Similarly, GzmA is shown to be adjacent to the nucleus of treated monocytes as seen by confocal microscopy. **(B)** Representative confocal microscopy image showing GzmA in red and DAPI (nuclear stain) in blue. **(C)** Representative confocal microscopy images from three separate experiments showing GzmA in red, DAPI (nuclear stain) in blue, and BCG-GFP in green. Comparing 30 min and 2 h post-infection, GzmA-treated cells show lower bacterial burden. **(D, E)** Image analysis isolating BCG-GFP+ areas at 16 h post-infection: superimposition of areas where BCG is present and GzmA. GzmA and BCG are not found in the same cells as visually represented in images and measured in graphs by looking at integrated density signal of BCG and its overlap with GzmA **(E)** (mean, at least three independent experiments) (*p < 0.05, **p < 0.01, ***p < 0.001; ns, not significant).

There are conflicting reports as to whether GzmA elicits its physiological effect intracellularly or at the cell surface (10, 11). To investigate this possibility, we performed flow cytometry studies using both surface and intracellular staining. Anti-human GzmA-PE was used either with or without cell permeabilization reagents to investigate GzmA localization. After overnight infection and treatment, higher frequencies of monocytes internalized GzmA compared to the protein levels adhered to the cell surface (**Figure 3A**). This internalization is also evident by confocal microscopy as GzmA is detected adjacent and surrounding the nucleus (**Figure 3B**). To further explore the timing and localization of GzmA in relation to mycobacteria, we performed a series of immunocytochemistry experiments during infection. There was evidence of mycobacterial control as early as 2 h post-infection as seen in (**Figure 3C**) and quantified as integrated density of BCG-GFP comparing untreated cells to GzmA-WT and GzmA-S195A treated cells (**Figure 3D**). At 16 h post-infection it appears that the smaller proportions of cells that would become infected after GzmA treatment had not internalized GzmA (**Figure 3E**). The merged image on the left was split so that BCG-GFP^+^ areas could be isolated (center) and overlapped with GzmA signal (right). In the lower panel, integrated density analyses quantified the amount of GzmA signal withing BCG-GFP^+^ areas, which were minimal, and compared the GzmA^+^ areas, which had very little BCG-GFP signal. The visualization and quantification further confirmed that there is no overlap between infection and GzmA, hinting at the possibility that those cells that do not internalize GzmA appear to be more prone to higher bacterial burden.

### Global Proteomic Analysis Uncovers Several Differentially Abundant Proteins

To better understand the mechanism of mycobacterial growth inhibition, we employed proteomics to identify differentially abundant proteins using the two-dimensional difference gel electrophoresis (2D-DIGE) platform. We studied each subject’s monocytes with four experimental groups, each with its own CyDye label that Melanie software (21) utilized to understand which protein spots were upregulated or downregulated with BCG infection or GzmA treatment (**Supplementary Figure 3**). The four groups studied are summarized in **Table 1**: 1) Monocytes alone, 2) Monocytes treated with GzmA, 3) Monocytes infected with BCG, 4) Monocytes infected with BCG and treated with GzmA. We focused on spots that were differentially abundant (>1.3-fold change in Group 4 *vs.* Group 1/2/3) in cells from Group 4 (BCG+GzmA), while the other groups served as controls. An example of a matched spot of interest is spot #431, shown in **Supplementary Figure 3**, which was upregulated in Group 4 and not in the other groups. Next, MALDI-TOF studies were employed to identify the differentially abundant protein spots (**Table 2**). Of these spots, ten were found using the human Mascot database (22). As a validation of our method, we investigated the identity of a protein that was not differentially abundant, and we identified cytoskeletal protein Tubulin 5 (TBB5). To the best of our knowledge, TBB5 has not been associated with differential expression in monocytes following mycobacterial infection or GzmA treatment, corroborating our investigation.

**Table 1:** Groups for proteomic analysis.

Group #	Infection and/or Treatment
1	Media-rested Monocvtes (DN)
2	GzmA-treated Monocytes
3	BCG-infected Monocytes
4	BCG-infected and GzmA-treated Monocvtes (DP)

The four groups studied are summarized here: 1) Monocytes alone, 2) Monocytes treated with GzmA, 3) Monocytes infected with BCG, 4) Monocytes infected with BCG and treated with GzmA.

**Table 2:** Global proteomic analysis uncovers several differentially abundant proteins.

#	ID	UNIPROT #	MALDI Score	Fold change (DP *vs.* DN)	Fold change (BCG+GzmA *vs.* BCG)
431	FBF1	Q8TES7	74	2.16	1.82
273	ACTB/G	P60709	66	2.18	1.80
272	CH60	P10809	84	1.69	1.58
43	ATP5H	O75947	80	1.62	1.54
323	BIP	P11021	240	1.77	1.54
280	INVO	P07476	70	2.24	1.47
268	PDIA1	P07237	65	1.90	1.46
374	Endoplasmin	P14625	97	1.71	1.44
259	PDlA3	P30101	103	1.31	1.39
332	GLU2B	P14314	114	1.70	1.34
228	TBB5	P07437	122	1.02	1.01

After software analysis Melanie identified 18 protein spots differentially abundant in 2 subjects, 10 proteins were identified using MALDI-TOF. # is the matched spot number for these proteins across subjects, their identity, UNIPROT#, MALDI score (which marks confidence of identification; i.e. score of 60 is the chance that 1 in a million that protein identified is a random event), Fold change Double Positive group (DP) vs Double Negative (DN) (group 4 abundancy/group 1 abundancy), Fold change DP vs BCG (group 4 abundancy/group 3 abundancy).

### ER Stress Response and ATP Synthesis Are Involved in GzmA-Mediated Mycobacterial Control

To understand the relationship between the identified proteins and the growth inhibition displayed by GzmA, we used the Search Tool for the Retrieval of Interacting Genes/Proteins (STRING) (16) database since it interrogates protein interactions on a global scale including stable physical associations, transient binding, substrate chaining, and information relay (17). After analyzing the results of all discovered spots, we focused on the Gene Ontology (GO) category of STRING and selected those pathways with a False Discovery Rate (FDR) ≤0.001. We identified two pathways associated with GzmA-mediated inhibition of intracellular mycobacteria: 1) the response to endoplasmic reticulum (ER) stress, and 2) mitochondrial ATP synthesis coupled proton transport. Protein Disulfide Isomerase A1 and A3 (PDIA1 and PDIA3), Binding immunoglobulin Protein (BiP), and Endoplasmin (HSP90B1) were identified as part of the ER stress pathway (**Supplementary Figure 4**). For the ATP synthesis pathway, we probed the STRING database using proteins that had a fold change >1.5 by both Group 4/Group 3 and Group 4/Group 1. This search alone did not yield a pathway, so additional nodes were created in STRING (increased from 5 original nodes to 10 machine-generated nodes), which identified ATP5H as a key protein (**Supplementary Figure 5**). Importantly, both the ER stress response (23–26) and ATP production leading to P2X7 receptor activation (27–35) have been implicated in mycobacterial control, consistent with our findings.

### Differentially Abundant Proteins Discovered With 2D-DIGE Are Also Differentially Abundant as Measured With Quantitative Western Blot Analysis

**Figure 4:**
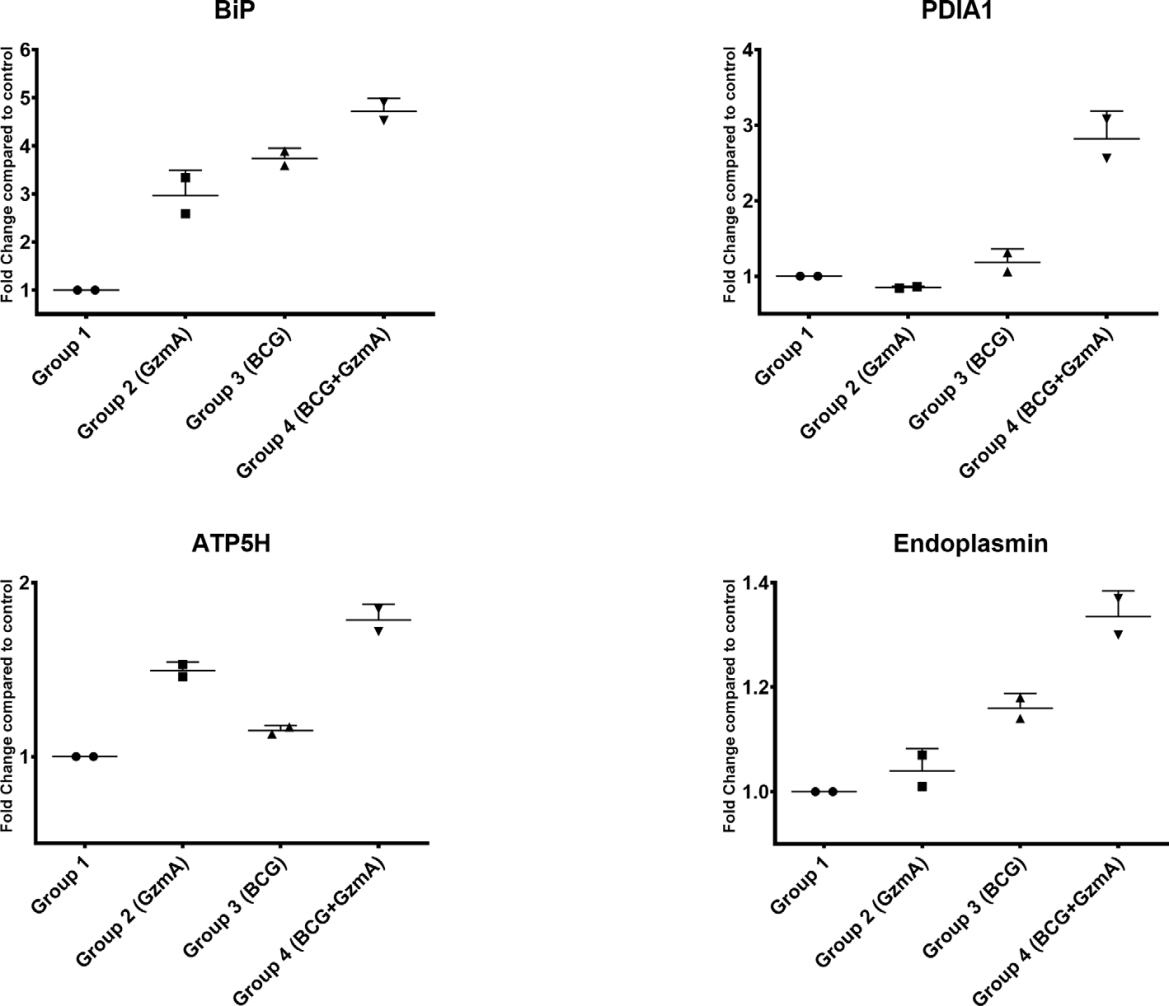
ER stress response and ATP producing proteins are involved in GzmA-mediated mycobacterial control. Graphs show four proteins that were selected from **Table 1**. Using Odyssey CLx near-infrared platform and total protein for normalization, data confirms 2D-DIGE results. Protein lysate obtained from 2 additional subjects that were not included in original 2D-DIGE analysis. Experiments done in triplicates per group, means ± SEM.

To validate our global proteomic data, we quantified the levels of three ER stress response proteins BiP, PDIA1, and Endoplasmin as well as the key protein involved in ATP production, ATP5H, in an independent set of volunteer samples. As displayed in (**Figure 4**) and as an example in (**Supplementary Figure 6**), the levels of these proteins were upregulated in Group 4 compared to the other control groups, confirming the increased protein levels observed by 2D-DIGE (**Table 2**).

## Discussion

γ_9_δ_2_ T cells are attractive candidates for novel vaccines against TB, because they are not MHC I restricted, and thus, can be broadly stimulated in the target population (36). GzmA is a key mediator employed by γ_9_δ_2_ T cells to control mycobacteria, but the mechanism by which it elicits an effect is unclear. While data in mice demonstrated that GzmA has no impact on TB control, it is important to note that γ_9_δ_2_ T cells are absent in mice and that TB pathology is markedly different than that in NHP or humans (37). Moreover, the mouse family of granzymes is larger than the human counterpart (9 *vs* 5 respectively), and biological redundancies between granzymes have been reported suggesting that mice are likely a poor model for studying the molecular mechanism of GzmA-mediated control of TB (38).

To perform global proteomic experiments, large quantities of human GzmA were required. Traditionally, NhGzmA is purified from the human NK cell line NK92MI encoding a constitutively expressed IL-2 transduced gene. However, this purification method requires more than four weeks to grow a sufficient number of cells to purify GzmA from their cytotoxic granules, and only yields approximately 50 µg of purified protein [data not shown and (14, 39)]. We demonstrate that RhGzmA phenocopies NhGzmA by assessing protein purity and formation of homodimer, enzymatic activity, and mycobacteria growth inhibition; our results also confirm previous reports that RhGzmA and NhGzmA have similar effects (10, 11). RhGzmA provides the key advantage of being able to generate large amounts of protein. Further, this recombinant construct may better reflect the native conformation than other commercially available or published constructs as the protein is expressed in a mammalian system to maintain full glycosylation of the protein.

GzmA-S195A induces mycobacterial killing similar to WT GzmA. As previously reported, GzmA-WT as well as GzmA-S195A are both able to mediate a synergic action with bacterial ligands to produce a pro-inflammatory profile in human monocytes (10), Thus, our studies confirm similar biological findings and create the opportunity for this pro-inflammatory phenotype to translate into an anti-mycobacterial role. As evidenced in (10), CD14 binding may be a key mediator for this action, and future studies will be required to investigate its role in the context of TB. Similarly, in a mouse model it has been shown that GzmA augments the response of plasmacytoid Dendritic Cells (pDC) through TLR9 to train adaptive immune cells (40), showing that GzmA could facilitate the activation of innate immune cells. More recently, it has been shown that GzmA cleaves Gasdermin B (GSDMB) to trigger pyroptosis of target cancer cells (41). However, this reported mechanism was triggered by 1) an intact active site, and 2) the use of perforin. Thus, while we did not investigate the role of GSDMB in our study, it is unlikely that the mycobactericidal role relies on this pathway. Similarly, it has been shown that patients and mice infected with arboviruses have increased levels of GzmA in the serum, and the use of a specific mouse GzmA inhibitor (Serpinb6b) can reverse the overactive inflammatory response (42); however, this mechanism was also reliant on an intact active site. The finding that GzmA-S195A also mediates mycobacterial inhibition opens the possibility that other key structural determinants are needed in this context such as homodimerization and/or glycosylation.

GzmA is internalized within one hour at steady state (11), and is detected inside cells after infection and treatment (**Figure 3**). Internalization of GzmA suggests that GzmA may be trafficked through an endocytic process after binding to a putative receptor. However, it is unclear whether intracellular GzmA is required for mycobacterial inhibition. In (11), GzmA was modified to more readily enter the cytosol of the target cell, and similar approaches could be explored. Other examples include site-directed mutagenesis of asparagine 170, which is glycosylated (43) and may affect the internalization rate by substituting it with glutamine. Cysteine residue at position 93, which is necessary for homodimerization, could be substituted to serine, as previously reported (44). Monomeric GzmA could lose the ability to bind to the putative receptor that is necessary for internalization, and/or GzmA ability to bind to bacteria and LPS as shown in (10).

Data indicate that an unbiased global proteomic analysis in primary cells is critical for uncovering novel substrates, since cell lines may identify substrates that lack biological relevance (45–47). Accordingly, when we compared our GzmA substrate data generated from primary human cells with previous reports that used cell lines, we found no information that would inform our studies (46, 47). There is evidence that GzmA is selective in the protein substrates that it cleaves due to its homodimeric configuration, and further, GzmA may only use a few substrates *in vivo* emphasizing the importance of the model system employed (44). Our model uses primary monocytes infected with mycobacteria and cells that are treated with physiological amounts of GzmA. As shown in (11), sub-micromolar concentrations of GzmA do not mediate pro-apoptotic events, but instead promote a different phenotype characterized with pro-inflammatory sequelae in human monocytes. Our studies were conducted at less than 200 nM concentrations, not noting an advantage of using higher concentrations of GzmA. Thus, our model may more closely resemble the natural immunological response after Mtb infection of alveolar macrophages (48). Future experiments are required to discern whether primary alveolar macrophages infected with Mtb recapitulate our findings. Our experiments were performed using mycobacteria BCG strain as we have previously shown that γ_9_δ_2_ T cells inhibit intracellular replication of Mtb as well as BCG through GzmA, and BCG has the advantage of being less biohazardous for our proteomic strategy (5).

**Figure 5:**
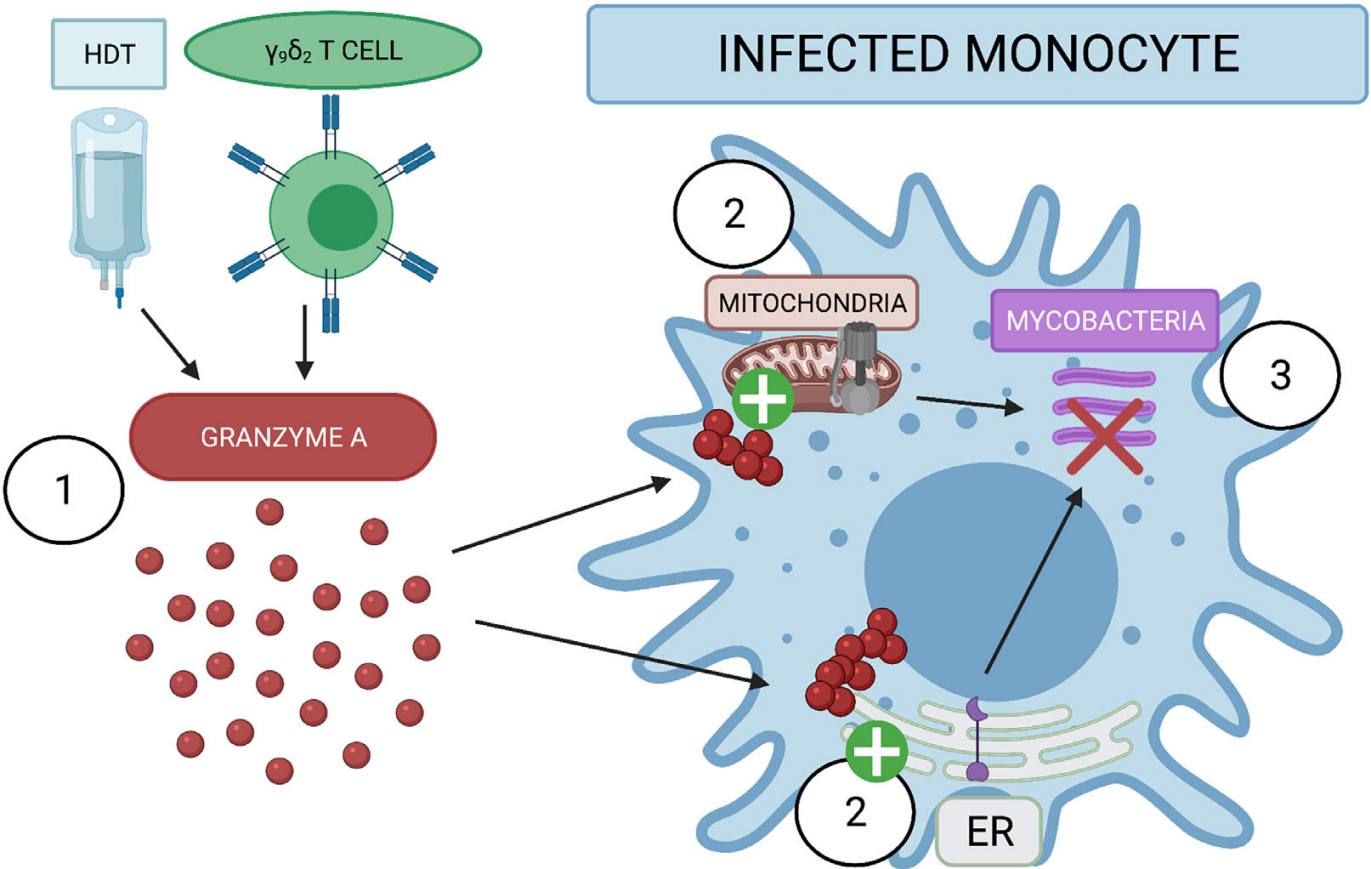
Extracellular GzmA added to infected monocyte induce mycobacterial inhibition by activating ER stress response and ATP producing proteins. (1) GzmA protein is released into the extracellular environment of infected monocytes either as an Host Directed Therapy (HDT) or from secretion of γ_9_δ_2_. GzmA is internalized and does not appear to cleave any cellular substrate. (2) GzmA instead activates ATP producing proteins and the ER stress response to induce (3) the inhibition of intracellular mycobacterial growth. Created with BioRender.com.

The pathways uncovered in our proteomic analysis are supported by previous data regarding host control of mycobacteria. For example, the ER stress response has been associated with M1 macrophage polarization, which leads to better host control of Mtb infection (26). While the precise mechanism has yet to be elucidated, this may be due to stimulation of the TLR2 pathway which triggers fusion of the lysosomal compartment with Mtb-containing phagosomes and induction of iNOS. Moreover, preliminary data indicate that antibiotics that induce ER-stress mediated autophagy, such as thiostrepton, are potential therapies for recently infected patients (23, 24). Thus, further studies are required to understand the potential treatment window in NHP. ATP-producing proteins such as ATP5H may help infected cells during energy-intensive processes such as protein production, protein folding, and cell metabolism (49). Future studies will investigate the ATP levels of GzmA-treated and infected monocytes; mycobacteria has been shown to reprogram host cell metabolism as shown in (50–52), and it is possible that GzmA is capable of switching cellular metabolism to favor the host. Moreover, the P2X7 receptor was recently shown to be important for mycobacterial control (27–35). This purinergic channel senses extracellular ATP and the cell responds to this stimulus by activating an inflammatory response and phagosome-lysosomal maturation. Further studies will be required to investigate the concentration of extracellular ATP and the involvement of the P2X7 receptor. As summarized in **Figure 5**, GzmA either used as Host Directed Therapy (HDT) or secreted from activated γ_9_δ_2_ T cells is internalized inside monocytes. In turn the treated monocyte activates the production of ATP producing proteins and the ER stress response pathway, leading to the inhibition of the intracellular mycobacterial growth. While these two pathways have been previously identified as important for mycobacterial control, to the best of our knowledge this is the first time that GzmA has been reported to induce these pathways for mycobacterial control and pathogen immunity. Future studies will investigate the necessity of these pathways for the induction of mycobacterial inhibition through gene alterations and pharmaceutical interventions to better describe GzmA mechanistic effects. These studies could lead to novel targets for host-directed therapies, as well as detection of important immune markers during future vaccine trials. Together, these strategies will contribute to the world health community’s goal of TB eradication.

## Data Availability Statement

The original contributions presented in the study are included in the article/**Supplementary Material**. Further inquiries can be directed to the corresponding author.

## Ethics Statement

The studies involving human participants were reviewed and approved by Institutional Review Board, Saint Louis University. The patients/participants provided their written informed consent to participate in this study.

## Author Contributions

VR, DW, CE, MX, RE, and DH designed key experiments. VR and DW performed global proteomics experiments. VR, NP, CE, MX, and DH designed GzmA-S195A, and VR tested enzymatically inactive GzmA. VR, RE, and DH designed confocal microscopy experiments, and VR executed them. MW and NB collaborated by providing key reagents, guidance through execution, and feedback on major experiments. All authors reviewed draft before submission. All authors contributed to the article and approved the submitted version.

## Funding

Research reported in this publication was supported by the National Heart, Lung, And Blood Institute under Award Number F30HL151136 to VR and National Institute of Allergy and Infectious Diseases of the National Institutes of Health under Award Number R01AI048391 to DH. NP was in part supported by a grant (R01 HL150146) from the National Heart, Lung and Blood Institute.

## Author Disclaimer

The content is solely the responsibility of the authors and does not necessarily represent the official views of the National Institutes of Health.

## Conflict of Interest

The authors declare that the research was conducted in the absence of any commercial or financial relationships that could be construed as a potential conflict of interest.

## Publisher’s Note

All claims expressed in this article are solely those of the authors and do not necessarily represent those of their affiliated organizations, or those of the publisher, the editors and the reviewers. Any product that may be evaluated in this article, or claim that may be made by its manufacturer, is not guaranteed or endorsed by the publisher.

## Supplementary Material

Supplementary Figure 1 RhGzmA acts independent of endotoxin. RhGzmA heated at 95°C for 10 min loses inhibitory activity, and RhGzmA passed through Endotrap column retains it. (n=4 in at least two independent experiments. Mean and SEM, paired t test).

Supplementary Figure 2 RhGzmA-S195A protein is pure and is detectable by western blot. Transient transfection of HEK293T cells expressing plasmid for RhGzmA-S195A yields a pure product without co-purification of other proteins. Anti-human GzmA binds to RhGzmA-S195A like RhGzmA-WT (**Figure 1A**) with a preference for the homodimer (Non-Reduced) conformation.

Supplementary Figure 3 Workflow diagram of global proteomic experiment and rationale for protein of interest. PBMC were treated or not with GzmA, and infected or not with BCG for 36 h. Cells were then washed with PBS, lysed with protease and phosphatase inhibitors, and downstream 2D-DIGE workflow. Example of matched spot #431. Melanie software analysis discovered this protein spot that was differentially abundant only in group 4 (BCG-infected, GzmA-treated monocytes) in two volunteers. Each labeled spot was normalized with CyDye2 labeling.

Supplementary Figure 4 STRING-DB enrichment pathways highlights ER stress response pathway involved in GzmA-mediated mycobacterial control. STRING database enriches protein for their relation to each other by known literature reports. Response to ER stress was upregulated when all the 10 proteins were interrogated.

Supplementary Figure 5 STRING-DB enrichment pathways highlights ATP synthesis pathway involved in GzmA-mediated mycobacterial control. STRING database enriches protein for their relation to each other by known literature reports. ATP synthesis was found after interrogating proteins with ≥1.5-fold change paired with machine-generated additional nodes.

Supplementary Figure 6 Example of BiP protein validated by Odyssey platform (quantitative western blot). On the left, protein was normalized using total protein stain, while on the right software analyzed signal and validated upregulation in BCG + GzmA group.
