# Supplementary figures and images for: Granzyme A Produced by γ9δ2 T Cells Activates ER Stress Responses and ATP Production, and Protects Against Intracellular Mycobacterial Replication Independent of Enzymatic Activity
Source: Front Immunol. 2021 Aug 3;12:712678. doi: 10.3389/fimmu.2021.712678 (PMC8368726; doi:10.3389/fimmu.2021.712678)

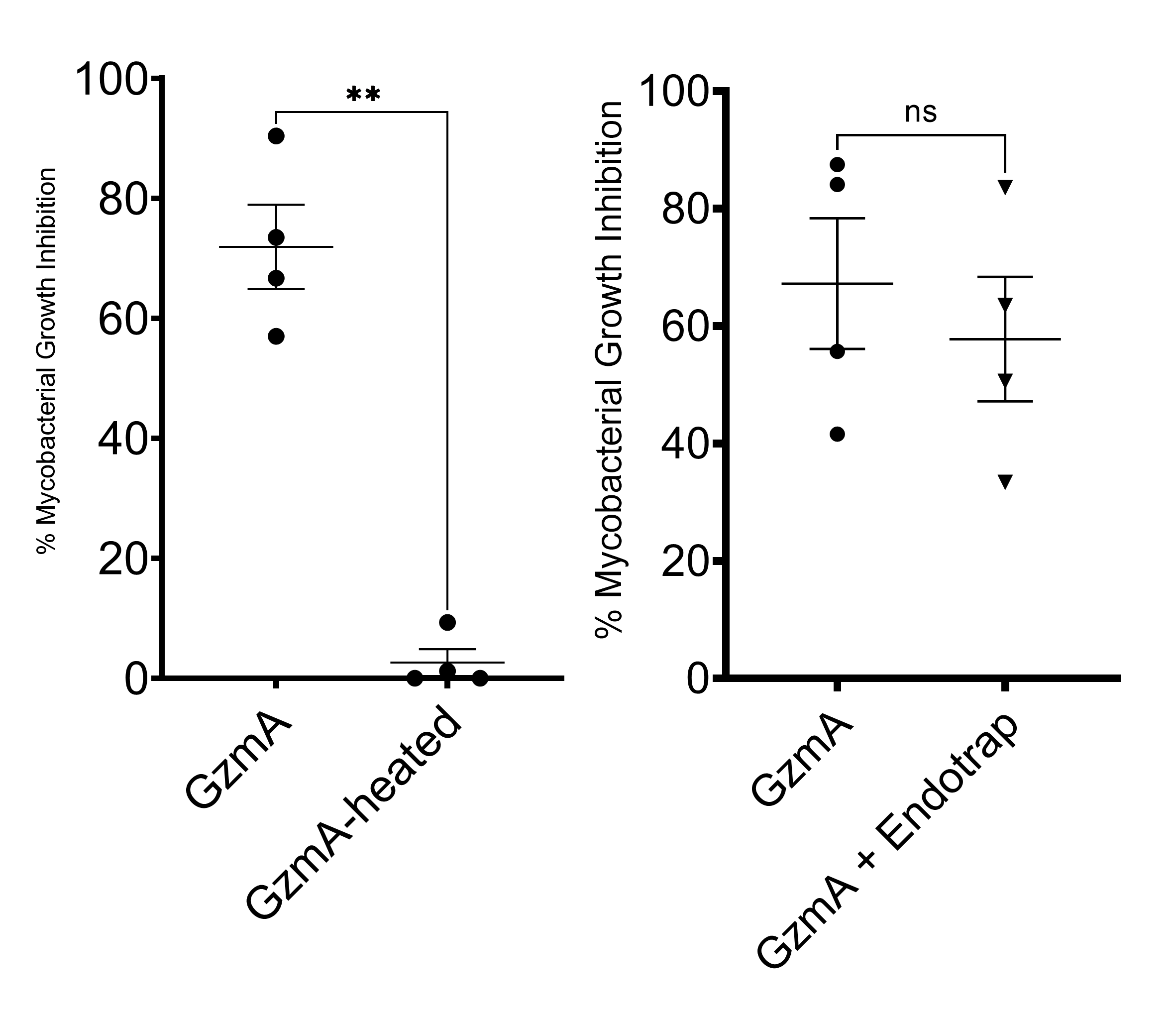

Supplement: Supplementary Figure 1 — RhGzmA acts independent of endotoxin. RhGzmA heated at 95°C for 10 min loses inhibitory activity, and RhGzmA passed through Endotrap column retains it. (n=4 in at least two independent experiments. Mean and SEM, paired t test). [file Image_1.tif]

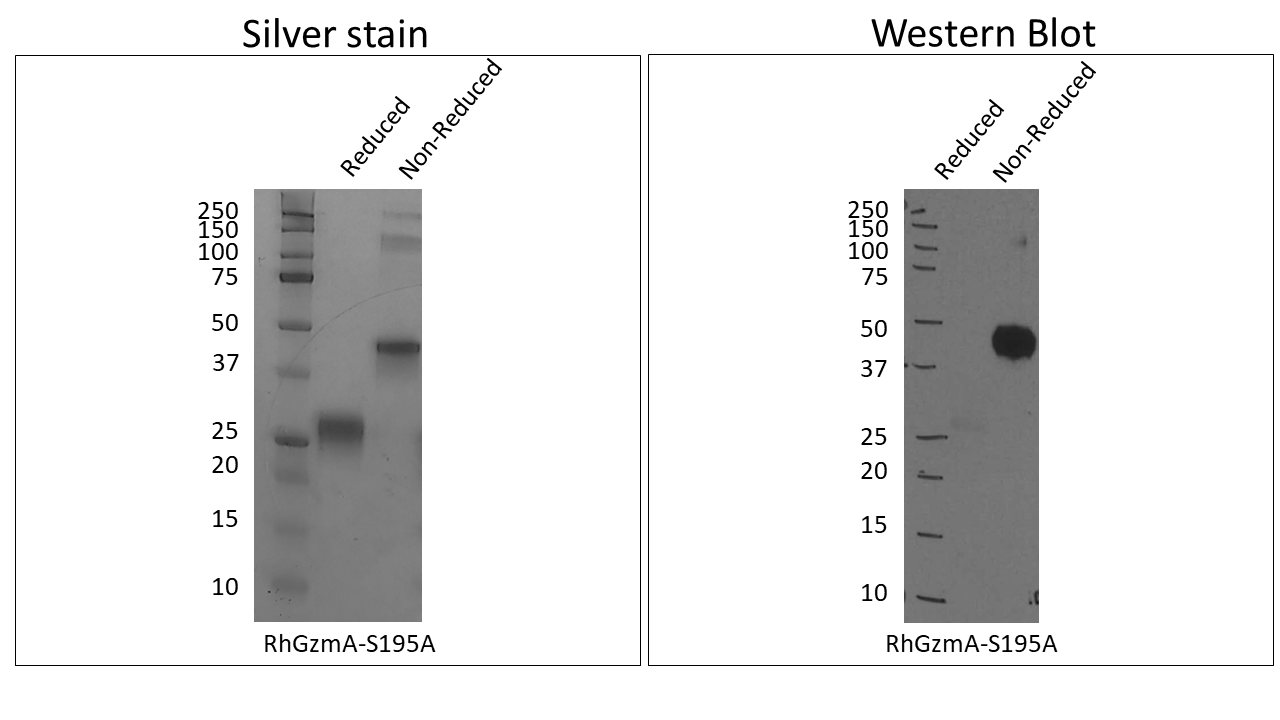

Supplement: Supplementary Figure 2 — RhGzmA-S195A protein is pure and is detectable by western blot. Transient transfection of HEK293T cells expressing plasmid for RhGzmA-S195A yields a pure product without co-purification of other proteins. Anti-human GzmA binds to RhGzmA-S195A like RhGzmA-WT (Figure 1A) with a preference for the homodimer (Non-Reduced) conformation. [file Image_2.tif]

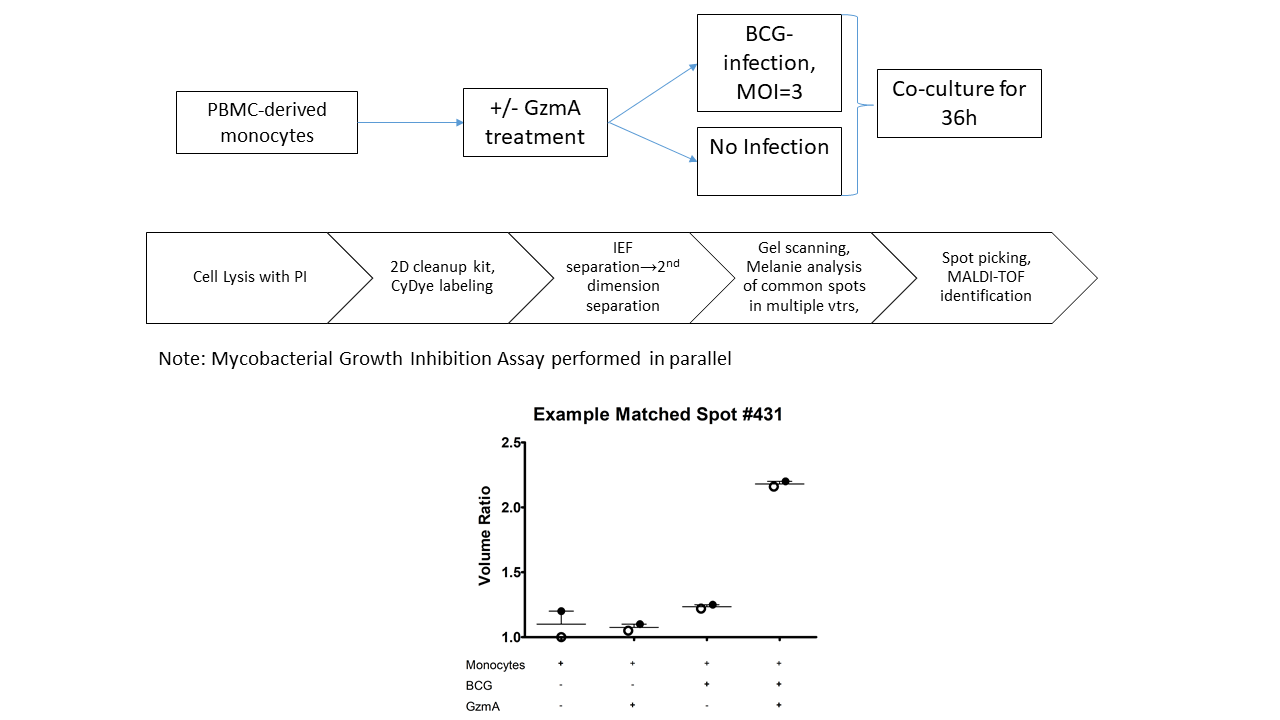

Supplement: Supplementary Figure 3 — Workflow diagram of global proteomic experiment and rationale for protein of interest. PBMC were treated or not with GzmA, and infected or not with BCG for 36 h. Cells were then washed with PBS, lysed with protease and phosphatase inhibitors, and downstream 2D-DIGE workflow. Example of matched spot #431. Melanie software analysis discovered this protein spot that was differentially abundant only in group 4 (BCG-infected, GzmA-treated monocytes) in two volunteers. Each labeled spot was normalized with CyDye2 labeling. [file Image_3.tif]

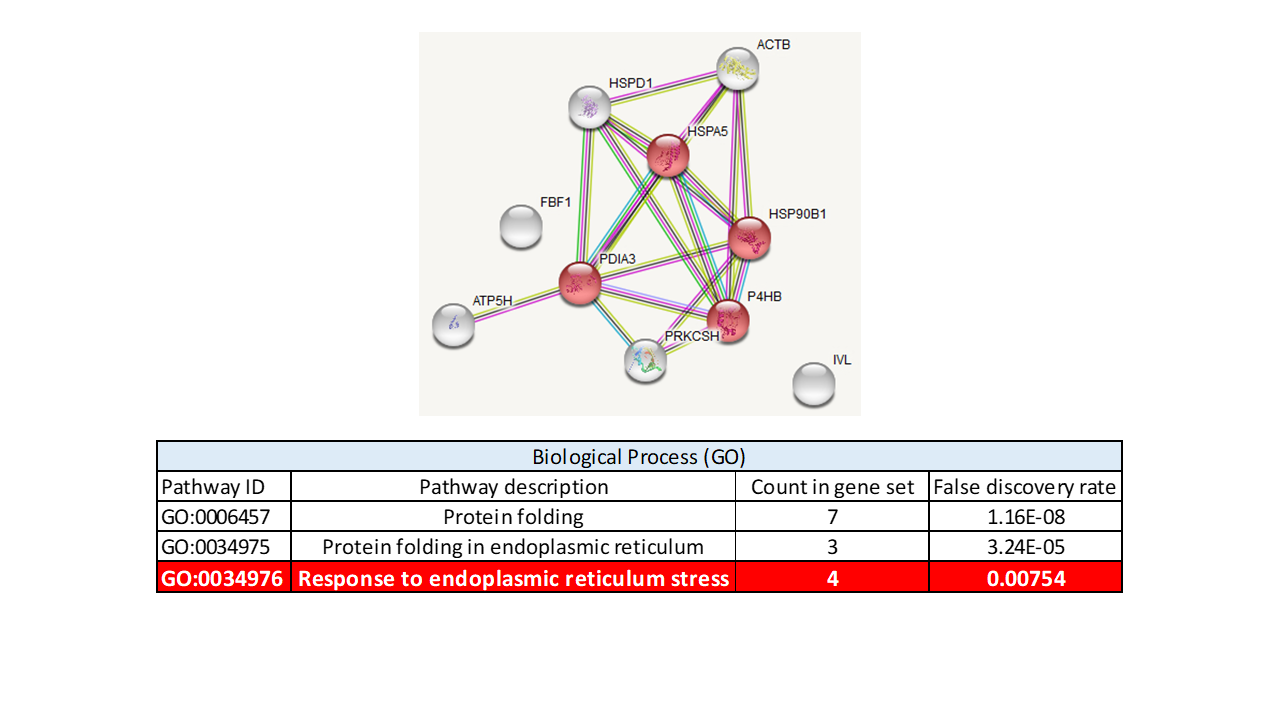

Supplement: Supplementary Figure 4 — STRING-DB enrichment pathways highlights ER stress response pathway involved in GzmA-mediated mycobacterial control. STRING database enriches protein for their relation to each other by known literature reports. Response to ER stress was upregulated when all the 10 proteins were interrogated. [file Image_4.tif]

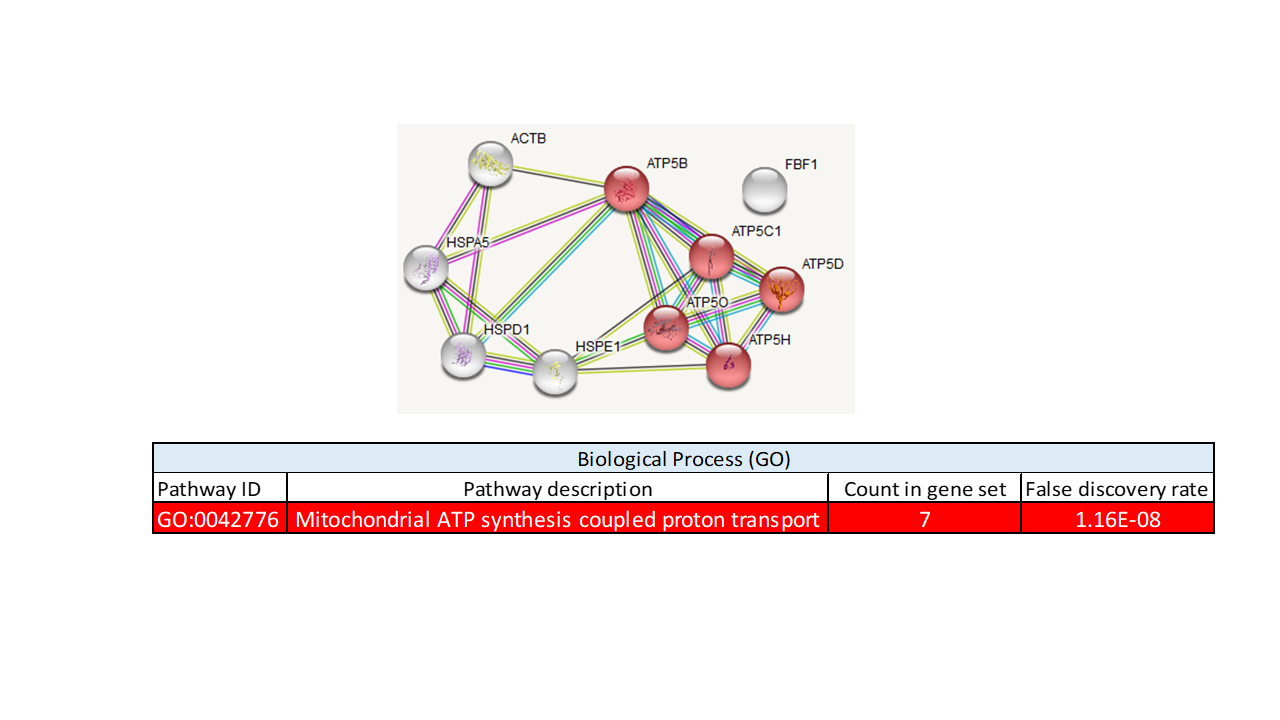

Supplement: Supplementary Figure 5 — STRING-DB enrichment pathways highlights ATP synthesis pathway involved in GzmA-mediated mycobacterial control. STRING database enriches protein for their relation to each other by known literature reports. ATP synthesis was found after interrogating proteins with ≥1.5-fold change paired with machine-generated additional nodes. [file Image_5.tif]

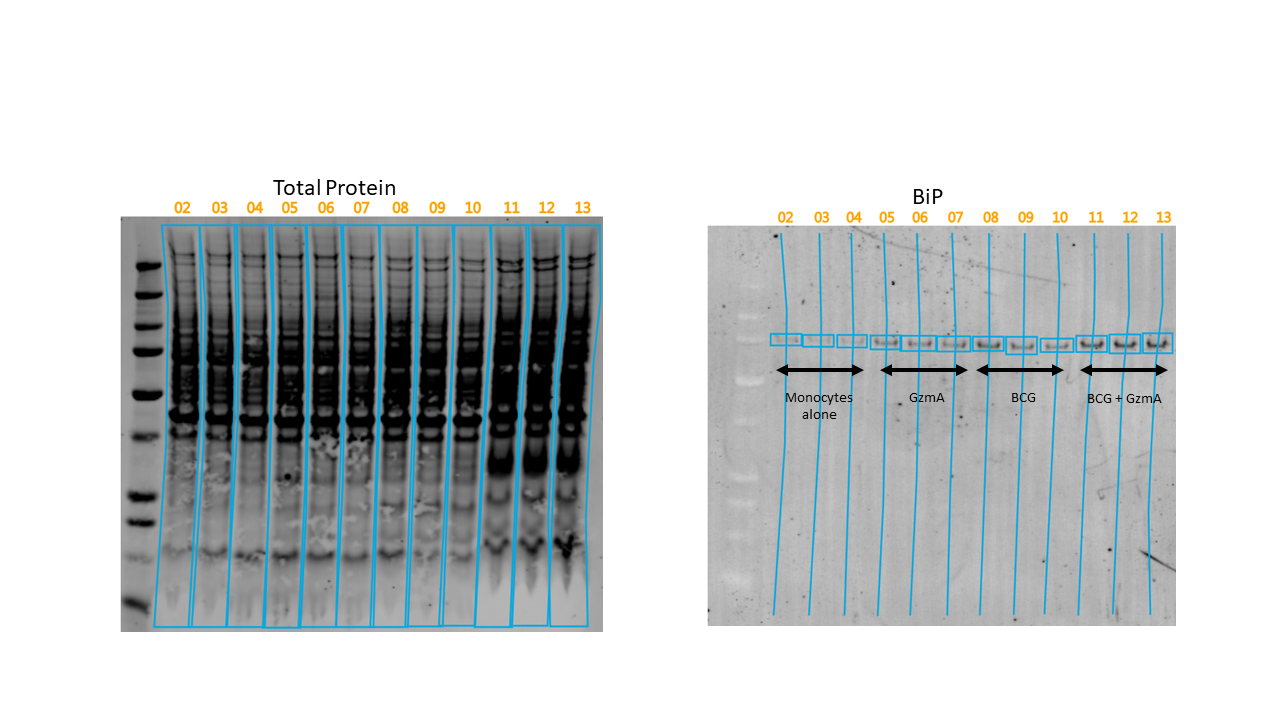

Supplement: Supplementary Figure 6 — Example of BiP protein validated by Odyssey platform (quantitative western blot). On the left, protein was normalized using total protein stain, while on the right software analyzed signal and validated upregulation in BCG + GzmA group. [file Image_6.tif]
